# The structure of Jann_2411 (DUF1470) from *Jannaschia* sp. at 1.45 Å resolution reveals a new fold (the ABATE domain) and suggests its possible role as a transcription regulator

**DOI:** 10.1107/S1744309109025196

**Published:** 2009-10-27

**Authors:** Constantina Bakolitsa, Alex Bateman, Kevin K. Jin, Daniel McMullan, S. Sri Krishna, Mitchell D. Miller, Polat Abdubek, Claire Acosta, Tamara Astakhova, Herbert L. Axelrod, Prasad Burra, Dennis Carlton, Hsiu-Ju Chiu, Thomas Clayton, Debanu Das, Marc C. Deller, Lian Duan, Ylva Elias, Julie Feuerhelm, Joanna C. Grant, Anna Grzechnik, Slawomir K. Grzechnik, Gye Won Han, Lukasz Jaroszewski, Heath E. Klock, Mark W. Knuth, Piotr Kozbial, Abhinav Kumar, David Marciano, Andrew T. Morse, Kevin D. Murphy, Edward Nigoghossian, Linda Okach, Silvya Oommachen, Jessica Paulsen, Ron Reyes, Christopher L. Rife, Natasha Sefcovic, Henry Tien, Christine B. Trame, Christina V. Trout, Henry van den Bedem, Dana Weekes, Aprilfawn White, Qingping Xu, Keith O. Hodgson, John Wooley, Marc-André Elsliger, Ashley M. Deacon, Adam Godzik, Scott Lesley, Ian A. Wilson

**Affiliations:** a Joint Center for Structural Genomics, http://www.jcsg.org, USA; b Program on Bioinformatics and Systems Biology, Burnham Institute for Medical Research, La Jolla, CA, USA; c Wellcome Trust Sanger Institute, Wellcome Trust Genome Campus, Hinxton CB10 1SA, England; d Stanford Synchrotron Radiation Lightsource, SLAC National Accelerator Laboratory, Menlo Park, CA, USA; e Protein Sciences Department, Genomics Institute of the Novartis Research Foundation, San Diego, CA, USA; f Center for Research in Biological Systems, University of California, San Diego, La Jolla, CA, USA; g Department of Molecular Biology, The Scripps Research Institute, La Jolla, CA, USA; h Photon Science, SLAC National Accelerator Laboratory, Menlo Park, CA, USA

**Keywords:** structural genomics, environmental stress, domains of unknown function, Pfam, bound metal identification

## Abstract

The crystal structure of Jann_2411 from *Jannaschia* sp. strain CCS1, a member of the Pfam PF07336 family classified as a domain of unknown function (DUF1470), was solved to a resolution of 1.45 Å by multiple-wavelength anomalous dispersion (MAD). This protein is the first structural representative of the DUF1470 Pfam family. Structural analysis revealed a two-domain organization, with the N-terminal domain presenting a new fold called the ABATE domain that may bind an as yet unknown ligand. The C-terminal domain forms a treble-clef zinc finger that is likely to be involved in DNA binding. Analysis of the Jann_2411 protein and the broader ABATE-domain family suggests a role as stress-induced transcriptional regulators.

## Abstract 2

The crystal structure of the first representative of the Pfam PF07336 (DUF1470) family reveals a two-domain organization that contains a new fold, termed the ABATE domain, at the N-terminus and a treble-clef zinc finger that is likely to bind DNA at the C-terminus.

## 1. Introduction

The complete genome sequences of hundreds of organisms are now known, each of which contains thousands of genes that have evolved to create the bewildering diversity of life. To understand this com­plexity at the molecular level requires the investigation of the function and structure of a vast number of proteins. A major goal of the Protein Structure Initiative (PSI; http://www.nigms.nih.gov/Initiatives/PSI/) is to expand our knowledge of the protein universe by solving the structures of representative members of large as yet uncharacterized protein families. The Pfam database (Finn *et al.*, 2008 ▶) contains over 2000 such families, termed domains of unknown function (DUFs), and understanding their structure will help to elucidate their function. Thus, to extend the structural coverage of proteins with uncharacterized biological function, we targeted the Pfam protein family DUF1470 and have determined the structure of the *Jann_2411* gene product from *Jannaschia* sp. strain CCS1, an ecologically relevant marine proteobacterium found in coastal and open surface waters.

Jann_2411 has a molecular weight of 20.7 kDa (residues 1–187) and a calculated isoelectric point of 6.6 and its crystal structure was determined using the semiautomated high-throughput pipeline of the Joint Center for Structural Genomics (JCSG; http://www.jcsg.org; Lesley *et al.*, 2002 ▶) as part of the NIGMS Protein Structure Initiative. Structural analysis revealed a two-domain organization, with the N-terminal domain consisting of a new fold that we call the ABATE (for Alpha-Beta-hairpin-Alpha TandEm) domain and the C-terminal domain forming a treble-clef zinc finger that we have termed the CGNR zinc-finger domain after a characteristic sequence motif that is conserved in this family. Jan_2411 forms a dimer, with both monomers implicated in the formation of a putative DNA binding site, and analysis of its genomic context suggests a role for the ABATE-domain family in stress-induced transcriptional regulation.

## 2. Materials and methods

### 2.1. Protein production and crystallization

The gene encoding Jann_2411 (GenBank YP_510353.1, gi:89054902; Swiss-Prot Q28PN4) was amplified by polymerase chain reaction (PCR) from genomic DNA using *PfuTurbo* DNA polymerase (Stratagene) and primers (forward primer, 5′-ctgtacttccagggcATG­AATTTAGACAGTTATGAAAGAACCG-3′; reverse primer, 5′-aa­ttaagtcgcgttaTGTTGCACGACGCTCGCGAAACGC­GGCG-3′; the target sequence is in upper case) corresponding to the predicted 5′ and 3′ ends. The PCR product was cloned into plasmid pSpeedET, which encodes an expression and purification tag followed by a tobacco etch virus (TEV) protease cleavage site (MGSDKIHHHH­HHENLYFQ/G) at the amino-terminus of the full-length protein. The cloning junctions were confirmed by DNA sequencing. Protein expression was performed in a selenomethionine-containing medium, with suppression of normal methionine synthesis, using the *Escherichia coli* strain GeneHogs (Invitrogen). At the end of fermentation, lysozyme was added to the culture to a final concentration of 250 µg ml^−1^ and the cells were harvested. After one freeze–thaw cycle, the cells were homogenized in lysis buffer [50 m*M* HEPES pH 8.0, 50 m*M* NaCl, 10 m*M* imidazole, 1 m*M* Tris(2-carboxy­ethyl)phos­phine hydrochloride (TCEP)] and passed through a Microfluidizer (Microfluidics). The lysate was clarified by centrifugation at 32 500*g* for 30 min and loaded onto nickel-chelating resin (GE Healthcare) pre-equilibrated with lysis buffer. The resin was washed with wash buffer [50 m*M* HEPES pH 8.0, 300 m*M* NaCl, 40 m*M* imidazole, 10%(*v*/*v*) glycerol, 1 m*M* TCEP] and the protein was eluted with elution buffer [20 m*M* HEPES pH 8.0, 300 m*M* imidazole, 10%(*v*/*v*) glycerol, 1 m*M* TCEP]. The eluate was buffer-exchanged with HEPES crystallization buffer (20 m*M* HEPES pH 8.0, 200 m*M* NaCl, 40 m*M* imidazole, 1 m*M* TCEP) using a PD-10 column (GE Healthcare) and treated with 1 mg TEV protease per 15 mg eluted protein. The digested eluate was passed over nickel-chelating resin (GE Healthcare) pre-equilibrated with HEPES crystallization buffer and the resin was washed with the same buffer. The flowthrough and wash fractions were combined and concentrated to 12.5 mg ml^−1^ by centrifugal ultrafiltration (Millipore) for crystallization assays. Jann_2411 was crystallized using the nanodroplet vapor-diffusion method (Santarsiero *et al.*, 2002 ▶) with standard JCSG crystallization protocols (Lesley *et al.*, 2002 ▶). Sitting drops composed of 200 nl protein solution mixed with 200 nl crystallization solution were equilibrated against a 50 µl reservoir at 277 K for 15 d prior to harvest. Initial screening for diffraction was carried out using the Stanford Automated Mounting system (SAM; http://smb.slac.stanford.edu/facilities/hardware/SAM/UserInfo; Cohen *et al.*, 2002 ▶) at the Stanford Synchrotron Radiation Lightsource (SSRL, Menlo Park, California, USA). The crystallization reagent consisted of 1.4 *M* sodium acetate and 0.1 *M* sodium cacodylate pH 6.5. A rod-shaped crystal of approximate dimensions 150 × 50 × 50 µm was harvested for data collection. Glycerol was added to the crystal as a cryoprotectant to a final concentration of 20%(*v*/*v*). The diffraction data were indexed in monoclinic space group *C*2 (Table 1 ▶). The oligomeric state of Jann_2411 in solution was determined using a 1 × 30 cm Superdex 200 size-exclusion column (GE Healthcare) coupled with miniDAWN static light scattering and Optilab differential refractive index detectors (SEC/SLS; Wyatt Technology). The mobile phase consisted of 20 m*M* Tris pH 8.0, 150 m*M* NaCl, and 0.02%(*w*/*v*) sodium azide. The molecular weight was calculated using *ASTRA* 5.1.5 software (Wyatt Technology).

### 2.2. Data collection, structure solution and refinement

Multiple-wavelength anomalous diffraction (MAD) data were collected at SSRL on beamline BL11-1 at wavelengths corresponding to the remote (λ_1_), inflection (λ_2_) and peak (λ_3_) of a selenium MAD experiment. The data sets were collected at 100 K with a MAR Mosaic 325 mm CCD detector (Rayonix, Evanston, Illinois, USA) using the *Blu-Ice* data-collection environment (McPhillips *et al.*, 2002 ▶). The MAD data were integrated and reduced using *XDS* and scaled with *XSCALE* (Kabsch, 1993 ▶). Initial substructure solution was performed with *SHELX* (Sheldrick, 2008 ▶) and the phases were refined with *SOLVE* (Terwilliger & Berendzen, 1999 ▶), with a mean figure of merit of 0.38 (0.59–2.0 Å) with two selenium sites. Density modification with *RESOLVE* (Terwilliger, 2003 ▶) was followed by automated model building using *ARP*/*wARP* (Cohen *et al.*, 2004 ▶). Model completion and refinement were performed with *Coot* (Emsley & Cowtan, 2004 ▶) and *REFMAC* 5.5 (Winn *et al.*, 2003 ▶) using the remote (λ_1_) data. The refinement included phase restraints from *SOLVE* and TLS refinement with four TLS groups per chain. Data-collection and refinement statistics are summarized in Table 1 ▶.

### 2.3. Identification of metal-binding sites

X-ray fluorescence emission peaks for selenium, arsenic, zinc and nickel were observed when the crystal was excited with X-rays 500 eV above the Se edge on SSRL beamline 11-1. In order to determine the identity of the metals at the individual sites in the structure, four additional data sets were collected on SSRL beamline 1-5. These data sets were collected to 2.9 Å resolution at wavelengths of 1.278, 1.292, 1.480 and 1.497 Å, which are above and below the zinc and nickel absorption edges. Data statistics are described in Table 2 ▶. Anomalous difference Fourier maps were calculated for each wavelength using the density-modified experimental MAD phases. The large changes in peak heights across the zinc or nickel absorption edge clearly showed that one site contained zinc and the other contained nickel. The integrated peak heights at the metal sites are shown with the peak heights for the selenium and sulfur sites as a reference in Table 3 ▶. The theoretical *f*′′ values at each wavelength are listed for comparison in Table 4 ▶.

### 2.4. Validation and deposition

**Figure 1:**
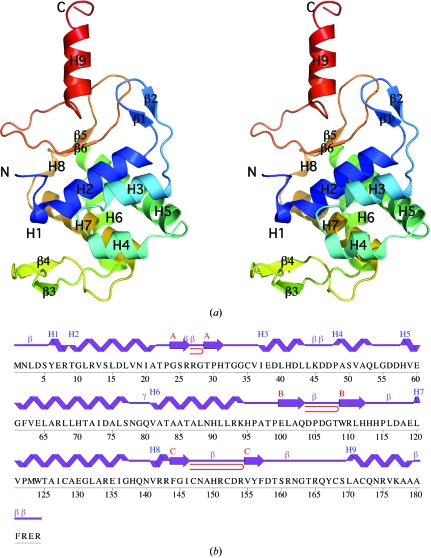
Crystal structure of Jann_2411 from *Jannaschia* sp. strain CCS1. (*a*) Stereo ribbon diagram of the Jann_2411 monomer color-coded from the N-terminus (blue) to the C-­terminus (red). Helices H1–H9 and β-strands (β1−β6) are indicated. (*b*) Diagram showing the secondary-structure elements of Jann_2411 superimposed on its primary sequence. The labeling of secondary-structure elements is in accord with *PDBsum* (http://www.ebi.ac.uk/pdbsum), where α-helices are labeled sequentially (H1, H2, H3 *etc*.), β-strands are labeled (A, B, C) according to the β-sheets to which they are assigned, β-turns and γ-turns are designated by their respective Greek letters (β, γ) and red loops indicate β-hairpins. For Jann_2411, the α-helices (H2–H7 and H9), 3_10_-helices (H1 and H8), β-sheets (A–C, comprising strands β1–β2, β3–β4 and β5–β6, respectively), β-turns (β) and β-hairpins are indicated.

The quality of the crystal structure was analyzed using the *JCSG Quality Control* server (http://smb.slac.stanford.edu/jcsg/QC). This server processes the coordinates and data using a variety of validation tools including *AutoDepInputTool* (Yang *et al.*, 2004 ▶), *MolProbity* (Davis *et al.*, 2007 ▶), *WHATIF* 5.0 (Vriend, 1990 ▶), *RESOLVE* (Terwilliger, 2003 ▶) and *MOLEMAN*2 (Kleywegt, 2000 ▶) as well as several in-house scripts and summarizes the output. Fig. 1 ▶(*b*) was adapted from an analysis using *PDBsum* (Laskowski *et al.*, 2005 ▶) and all other figures were prepared with *PyMOL* (DeLano Scientific). Atomic coordinates and experimental structure factors for Jann_2411 have been deposited in the PDB (http://www.pdb.org) under accession code 3h0n.

## 3. Results and discussion

### 3.1. Overall structure

The crystal structure of Jann_2411 (Fig. 1 ▶ *a*) was determined to 1.45 Å resolution using the MAD method. Data collection and refinement statistics are summarized in Table 1 ▶. The final model included one protomer (residues 1–184), three acetate molecules, two glycerol molecules, one zinc ion, one nickel ion, two sodium ions and 240 water molecules in the asymmetric unit. Arg185, Ala186 and Thr187 at the C-terminus and Gly0 remaining after the cleavage of the expression/purification tag at the N-terminus were disordered and not modeled. Poor electron density was observed for the side chains of Lys45, Arg161, Asn162, Lys177 and Arg182. The side chains of the following residues were modeled in two conformations: Ile37, Asp42, Arg67, Asp74, His90, Gln103, Leu116, Glu119, Leu120 and Met123. The Matthews coefficient (*V*_M_; Matthews, 1968 ▶) was 2.5 Å^3^ Da^−1^ and the estimated solvent content was 50.9%. The Ramachandran plot produced by *MolProbity* (Davis *et al.*, 2007 ▶) showed that 98.9% of the residues were in favored regions, with no outliers.

Jann_2411 belongs to the Pfam family known as DUF1470, which accounts for the entire length of the protein sequence. However, the structure shows that Jann_2411 is actually comprised of two domains (Figs. 1 ▶ *a* and 2 ▶ *a*). The first domain (residues 1–142) can be visualized as two subdomains (H2–H4, β1–β2 and H5–H7, β3–β4) that share similar topology and secondary-structure elements, namely a helix–β-­hairpin–helix motif (H2-β1-β2-H3 in the first subdomain; H6-β3-β4-H7 in the second subdomain), with an additional helix (H4 from the first subdomain and H5 from the second subdomain) linking the two motifs. We have therefore named this region the ABATE domain, representing the Alpha-Beta-hairpin-Alpha TandEm motif. The helices of the first subdomain (H2 and H3) are stacked per­pendicular to helices H5 and H7 which, together with H6, form a helical bundle capped on one end by the 3_10_-helix H8 and on the other by helix H4. In both subdomains, the β-hairpin is oriented orthogonally with respect to the long axes of the helices in the ABATE motif. Superposition of the two ABATE motifs (residues 9–46 for ABATE1 and 80–139 for ABATE2) results in an r.m.s.d. of 3.2 Å over 27 residues (3% identity), which is non-significant. Additionally, sequence alignment shows little residue conservation in this domain, with no strictly conserved residues observed between representative ABATE-family sequences (Fig. 3 ▶), suggesting that this domain evolved as a single unit as opposed to the gene-duplication event that might be suggested by the presence of two ABATE motifs.

**Figure 2:**
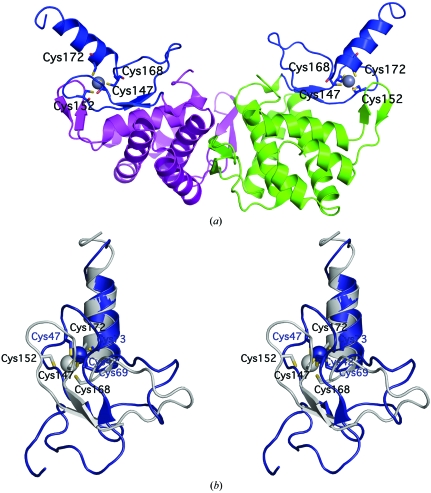
The C-terminal domain of Jann_2411 forms a zinc finger. (*a*) Ribbon representation of the Jann_2411 dimer. The zinc-finger domains are depicted in blue, the N-terminal domains are depicted in magenta and green and the zinc ions are shown as gray spheres. The zinc-coordinating cysteines are shown in ball-and-stick representation and labeled. (*b*) Stereoview of the structural superposition of the C-terminal domain of Jann_2411 (PDB code 3h0n, residues 144–187, gray) and a PHD finger fragment from yeast Yng1 protein (PDB code 2jmi, residues 38–83, blue). Zinc ions are shown as spheres and side chains of coordinating residues are indicated.

**Figure 3:**
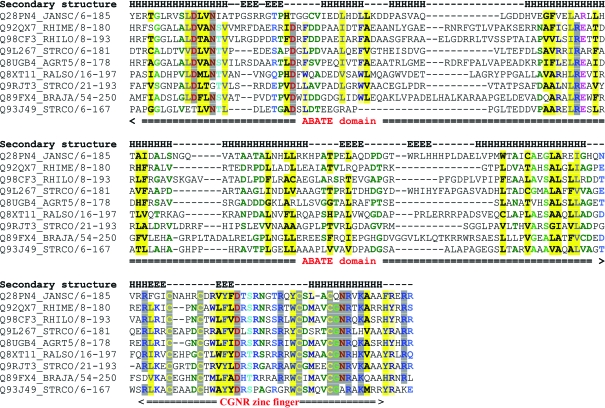
Multiple sequence alignment of Jann_2411 and representative ABATE-family sequences from related species. Sequences were chosen from the DUF1470 Pfam seed alignment. The alignment was derived from the Pfam full alignment. UniProt abbreviations are as follows: Q28PN4_JANSC, gene locus Jann_2411 from *Jannaschia* sp. (strain CCS1); Q92QX7_RHIME, gene locus R01166 from *Rhizobium meliloti*; Q98CF3_RHILO, gene locus mlr5173 from *R. loti*; Q9L267_STRCO, gene locus SCO1542 from *Streptomyces coelicolor*; Q8UGB4_AGRT5, gene locus Atu1124 from *Agrobacterium tumefaciens*; Q8XT11_RALSO, gene locus RSp0306 from *Ralstonia solonacearum*; Q9RJT3_STRO, gene locus SCO0403 from *S. coelicolor*; Q89FX4_BRAJA, gene locus bII6575 from *Bradyrhizobium japonicum*; Q93J49_STRCO, gene locus SCO3054 from *S. coelicolor*. Residues are colored by conservation using the *CHROMA* software with default settings (Goodstadt & Ponting, 2001 ▶).

The second domain (residues 143–187; H8, β5–β6, H9) forms a treble-clef zinc finger (Fig. 2 ▶). The zinc ion is coordinated by two cysteines (Cys147 and Cys152) from a loop termed the zinc knuckle, located between the strands of the third β-hairpin (β5–β6), and two cysteines from the N-terminus of helix H9 (Cys168 and Cys172) (Fig. 2 ▶ *a*). This arrangement of zinc-coordinating residues is typical of treble-clef zinc fingers (Grishin, 2001 ▶; Krishna *et al.*, 2003 ▶). Other strictly conserved residues (Fig. 3 ▶) in this domain include Asp158 and Arg175. A high degree of conservation is observed for a number of positively charged residues (Arg143, Arg161, Arg165, Lys177, Arg182 and Arg184), suggesting that this region could present a nucleic acid binding site. Furthermore, residues 146 (a hydrophobic residue) and 167 (an aromatic residue) are highly conserved (Fig. 3 ▶) and could intercalate between the DNA bases. Based on the most conserved motif found in the C-terminal α-helix in this family of proteins, we have named this domain the CGNR zinc finger. The actual amino-acid sequence in Jann_2411 is CQNR.

The above findings led to the re-evaluation of the Pfam DUF1470 family which, as a result of our study, will now be split into two entries in the next Pfam release (the current release is Pfam 23.0, July 2008). The original DUF1470 entry has been truncated and renamed to represent the ABATE domain, while a new Pfam family has been created for the C-­terminal CGNR zinc-finger domain called zf-­CGNR (Pfam accession PF11706).

### 3.2. Similarity to other proteins

*SCOP* classifies Jann_2411 as an α+β protein with an unusual fold (http://scop.mrc-lmb.cam.ac.uk/scop/data/scop.b.e.dda.b.b.b.html). A search with *FATCAT* (Ye & Godzik, 2004 ▶) using the N-terminal domain of Jann_2411 gave some hits involving helices H5–H7, but these structures [a three-helix bundle from a bacterial ATPase (PDB code 2v6y) and a histidine phosphotransferase domain (PDB code 1sr2)] involved less than half of the domain and displayed diverse functionalities. No single hit was found for the N-terminal domain in its entirety, leading us to propose that this domain represents a new fold. The C-terminal domain was structurally similar (main-chain r.m.s.d. of 2.5 Å over 40 residues with a sequence identity of 11%) to a plant homeodomain (PHD) finger from yeast (PDB code 2jmi), confirming the identity of this domain as a treble-clef zinc finger. Superposition of H9 onto the corresponding helix of the yeast structure revealed that the arrangement of the zinc ion and coordinating cysteines is conserved between the two structures (Fig. 2 ▶ *b*).

Analysis of the crystallographic packing of Jann_2411 using the *PISA* server (Krissinel & Henrick, 2007 ▶) and analytical size-exclusion chromatography in combination with static light scattering indicate that a dimer is the likely quaternary form. The crystallographic dimer interface mainly involves hydrophobic contacts from the second β-­hairpin (strands β3–β4), helices H6 and H7 and the intervening loops, and buries 990 Å^2^ of surface area per monomer (Fig. 2 ▶ *a*). This arrangement results in the formation of a deep cavity (∼2800 Å^3^ according to the *CastP* server; Binkowski *et al.*, 2003 ▶) along the dimer interface, delimited by the long loop connecting the last hairpin (strands β5–β6) and helix H9. However, sequence and structure conservation in this domain is very weak, making quaternary states difficult to infer for the rest of the family.

Treble-clef zinc fingers are usually incorporated into larger structures and are found in proteins with a wide range of functions, many of which involve transcriptional regulation (Grishin, 2001 ▶; Krishna *et al.*, 2003 ▶). *Jannaschia* sp. CCS1 is a member of the *Roseobacter* lineage, a taxon of marine bacteria. CCS1 is a phototroph that uses bacteriochlorophyll to harvest energy from light without the formation of oxygen. Genes predicted to have functional associations with Jann_2411 in the STRING database (http://string.embl.de) include a transmembrane protein of unknown function (Jann_2410) and the transcriptional regulator Jann_2412, a member of the *Asr* gene family. The *Asr* gene family is widespread in higher plants and most members of this family are up-regulated under a range of environmental stress conditions; their products are thought to function as transcriptional regulators (Frankel *et al.*, 2006 ▶).

Other members of this newly defined ABATE protein family are found in plant symbionts (*Rhizobium*, *Bradyrhizobium*) and plant pathogens (*Streptomyces*, *Ralstonia*, *Agrobacterium*); they are around 180 residues in length and also contain the newly designated zf-­CGNR (Pfam accession PF11706). Genome-location analysis of representative ABATE sequences shows co-occurrence with putative DNA-binding proteins, transcriptional regulators and membrane proteins implicated in ABC transport. In *Streptomyces*, several of the proteins co-occurring with ABATE homologs, such as sporulation-specific cell-division proteins and RNA polymerase sigma factors, are implicated in the control of aerial mycelium development (Dalton *et al.*, 2007 ▶; Gordon *et al.*, 2008 ▶) and are activated under conditions of cell-envelope stress, such as hyperosmolarity (Kormanec & Sevcikova, 2002 ▶).

A number of molecular mechanisms are shared between symbionts and pathogens, especially those involving host colonization and adaptation to a particular ecological niche (Hentschel *et al.*, 2000 ▶). In both instances, changes in ecological and host environments necessitate fast adaptation strategies on the part of the microorganism. Zinc fingers possess the functional versatility necessary for this adaptation and have been exploited may times by nature and by the pharmaceutical industry (Papworth *et al.*, 2006 ▶). The molecular function of the ABATE domain remains elusive. However, given the prediction that the C-terminal domain binds DNA, then the N-­terminal domain may allow the protein to act as a signal-dependent transcriptional regulator with the ABATE domain conferring sensitivity to some as yet undefined ligand. This combination of a DNA-binding domain with a ligand-sensing domain is a prevalent form of regulation of operons in bacteria, such as the lactose or arabinose operons (Anantharaman *et al.*, 2001 ▶). The likely dimeric nature of the protein and the long loop delimiting the suggested DNA-binding region hint at the possibility of an allosteric mechanism that might abolish DNA binding upon binding to an as yet unknown ligand. The LacI protein represents a canonical example for such an allosteric transition, with ligand-binding inducing a hinge-like motion that alters the relative subdomain orientations within the dimer, thereby changing DNA affinity (Lewis *et al.*, 1996 ▶). Many other similar examples exist, such as TetR (Henssler *et al.*, 2005 ▶; Premkumar *et al.*, 2007 ▶; Koclega *et al.*, 2007 ▶), all of which form dimers or higher order oligomers.

The availability of additional ABATE sequences and structures should shed light on the evolutionary history of this protein family. The information presented here, in combination with further biochemical and biophysical studies, should yield valuable insights into the functional role of Jann_2411. Models of Jann_2411 homologs can be accessed at http://www1.jcsg.org/cgi-bin/models/get_mor.pl?key=3h0nA.

Additional information about the protein described in this study is available from TOPSAN (Krishna *et al.*, 2010 ▶)  http://www.topsan.org/explore?PDBid=3h0n.

## 4. Conclusions

The first structural representative of the DUF1470 family revealed a two-domain organization, with the N-terminal domain presenting a new fold and the C-terminal domain consisting of a treble-clef zinc finger. The structure additionally allowed a re-evaluation of the Pfam signature and the Pfam assignment and suggests a role for this family in stress-induced transcriptional regulation.

## Supplementary Material

PDB reference: Jann_2411 from *Jannaschia* sp. strain CCS1, 3h0n, r3h0nsf

## Figures and Tables

**Table 1:** Summary of crystal parameters, data-collection and refinement statistics for Jann_2411 (PDB code 3h0n) Values in parentheses are for the highest resolution shell.

	λ_1_ MADSe	λ_2_ MADSe	λ_3_ MADSe
Data collection			
Space group	*C*2		
Unit-cell parameters (Å, °)	*a* = 77.75, *b* = 59.67, *c* = 57.82, β = 128.8		
Wavelength (Å)	0.9184	0.9792	0.9788
Resolution range (Å)	25.8–1.45 (1.49–1.45)	25.8–1.45 (1.49–1.45)	25.8–1.45 (1.49–1.45)
No. of observations	109293	108225	108208
No. of unique reflections	36254	36192	36235
Completeness (%)	99.2 (98.3)	99.1 (96.3)	99.1 (96.8)
Mean *I*/σ(*I*)	19.0 (4.1)	17.7 (3.6)	17.9 (3.4)
*R*_merge_ on *I*† (%)	4.1 (29.1)	4.4 (30.8)	4.7 (34.0)
Model and refinement statistics			
Resolution range (Å)	25.0–1.45		
No. of reflections (total)	36254		
No. of reflections (test)	1810		
Completeness (%)	99.1		
Data set used in refinement	λ_1_ MADSe		
Cutoff criterion	|*F*| > 0		
*R*_cryst_‡	0.140		
*R*_free_§	0.157		
Stereochemical parameters			
Restraints (r.m.s. observed)			
Bond angles (°)	1.44		
Bond lengths (Å)	0.015		
Average isotropic *B* value (Å^2^)	16.5¶		
ESU†† based on *R*_free_ (Å)	0.053		
Protein residues/atoms	184/1499		
Waters/other molecules	240/9		

† *R*_merge_ = 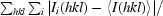 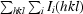.

‡ *R*_cryst_ = 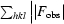 − 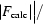 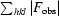, where *F*_calc_ and *F*_obs_ are the calculated and observed structure-factor amplitudes, respectively.

§ *R*_free_ is the same as *R*_cryst_ but for 5.0% of the total reflections that were chosen at random and omitted from refinement.

¶ This value represents the total *B* that includes TLS and residual *B* components.

†† Estimated overall coordinate error (Collaborative Computational Project, Number 4, 1994 ▶; Tickle *et al.*, 1998 ▶).

**Table 2:** Data-collection statistics for metal-site identification Values in parentheses are for the highest resolution shell.

	λ_4_ above Zn	λ_5_ below Zn	λ_6_ above Ni	λ_7_ below Ni
Wavelength (Å)	1.2782	1.2915	1.4795	1.4974
Resolution range (Å)	45.1–2.90 (2.98–2.90)	45.1–2.90 (2.98–2.90)	45.1–2.90 (2.98–2.90)	45.1–2.90 (2.98–2.90)
No. of observations	17029	16952	15853	15655
No. of unique reflections	4658	4658	4527	4489
Completeness (%)	99.7 (99.6)	99.8 (98.9)	97.0 (77.2)	96.1 (72.5)
Mean *I*/σ(*I*)	47.4 (32.8)	51.8 (35.7)	50.2 (28.9)	50.8 (27.1)
*R*_merge_ on *I*† (%)	2.9 (4.0)	2.2 (3.3)	2.3 (3.1)	2.2 (3.0)

† *R*_merge_ = 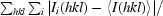 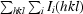.

**Table 3:** Anomalous difference Fourier integrated peak heights The maps were calculated with data from 20 to 2.9 Å. The signal listed is the value reported by *MAPMAN* (Kleywegt & Jones, 1996 ▶) after integration of a sphere of radius 2 Å around the atom center from the final refined model. Sulfur and selenium sites are listed to provide a reference for differences in scale between different maps.

Atom	λ_1_ MADSe	λ_4_ above Zn	λ_5_ below Zn	λ_6_ above Ni	λ_7_ below Ni
Se-1	11.26	2.96	1.57	2.93	3.13
Se-123a	21.11†	5.64	3.50	4.43	4.23
Se-123b	21.84†	5.91	3.60	4.47	4.30
Zn	15.69	17.43	2.92	3.97	4.11
Ni-a	5.07†	5.49	5.27	4.59	0.42
Ni-b	3.88†	3.07	2.84	3.34	0.39
S-35	0.25	0.70	1.02	1.58	1.84
S-128	0.48	1.10	1.30	2.12	2.30
S-147	3.44	2.51	1.86	2.21	2.72
S-152	2.44	2.88	1.48	2.35	2.03
S-168	3.23	3.55	1.45	2.38	2.79
S-172	5.47	5.11	2.29	2.59	2.77

† The Se atom from residue A123 and the Ni atom from residue A202 were modeled in alternate conformations and each partial occupancy site was integrated separately without overlap removal.

**Table 4:** Theoretical *f*′′ values at each wavelength Theoretical *f*′′ data were extracted from tables compiled by Ethan Merritt (http://skuld.bmsc.washington.edu/scatter/AS_periodic.html).

Data set	λ_1_ MADSe	λ_4_ above Zn	λ_5_ below Zn	λ_6_ above Ni	λ_7_ below Ni
Wavelength (Å)	0.9184	1.2782	1.2915	1.4795	1.4974
Energy (eV)	13500	9700	9600	8380	8280
Theoretical *f*′′ Se	3.37	0.81	0.83	1.06	1.08
Theoretical *f*′′ Zn	2.23	3.86	0.49	0.63	0.64
Theoretical *f*′′ Ni	1.75	3.05	3.10	3.87	0.48
Theoretical *f*′′ S	0.21	0.39	0.40	0.52	0.53
